# Evaluation of a Reduced Work Schedule and Housing Authority Employee Wellbeing

**DOI:** 10.3390/ijerph23081026

**Published:** 2026-08-06

**Authors:** Morgan A. Valley, Katie McMahon, Aurora Koren, Lorann Stallones

**Affiliations:** 1 Department of Environmental & Radiological Health Sciences, Colorado State University, Fort Collins, CO 80523, USA; 2 Department of Psychology, Colorado State University, Fort Collins, CO 80523, USA; katie.mcmahon@colostate.edu (K.M.); lorann.stallones@colostate.edu (L.S.); 3 Colorado School of Public Health, Colorado State University, Fort Collins, CO 80523, USA; aurora.koren@colostate.edu

**Keywords:** reduced work schedule, worker wellbeing, work stress, evaluation

## Abstract

**Highlights:**

**Public health relevance—How does this work relate to a public health issue?**

- Public housing employees work in challenging conditions with many stressors. Reduced work schedules are an organizational-level intervention with the potential to reduce the negative impacts of these stressors.
- Work stress, work overload, and work-to-non-work conflict contribute to burnout and impact mental and physical health, making workplace interventions that reduce these outcomes relevant to public health.

**Public health significance—Why is this work of significance to public health?**

- This study provides evidence that participation in a voluntary reduced work schedule was associated with lower work overload, an effect that was observed consistently across 18 months.
- Findings show that wellbeing benefits of a reduced work schedule program were not perceived as equally accessible across job roles, with client-facing and leadership staff describing greater difficulty disconnecting from work.

**Public health implications—What are the key implications or messages for practitioners, policymakers and/or researchers in public health?**

- For public sector and nonprofit organizations with demanding, client-facing work, reduced work schedules may be a sustainable strategy to protect employee wellbeing but should be paired with adequate staffing and explicit workload redistribution so benefits are spread across roles.
- Practitioners and researchers should consider how reduced schedule program design and job role shape outcomes, and future studies on reduced work schedules should incorporate baseline measures and comparison designs to strengthen causal inference about reduced schedules and worker wellbeing.

**Abstract:**

Background: Public housing employees work in challenging conditions with many stressors. A Colorado housing authority requested an evaluation of its voluntary reduced work schedule pilot program and its association with employee wellbeing. Previous research has shown promising short-term improvements in employee productivity and morale from reduced schedule programs, but researchers have questioned whether impacts on wellbeing could be sustained. The study aimed to evaluate if program participants and non-participants reported differences in wellbeing measures over 18 months post-implementation. Methods: A longitudinal survey based on the NIOSH WellBQ over three timepoints and a series of four focus groups were conducted with program participants and non-participants. Linear mixed modeling was used to test for differences between participant and non-participant wellbeing outcomes over time. Deductive thematic analysis was used to analyze focus group data. Results: Program participants reported significantly lower work overload than non-participants; differences in work stress and work-to-non-work conflict were smaller and did not reach statistical significance. There was no evidence that the magnitude of these differences changed across timepoints, although limited statistical power constrains interpretation of this finding. Qualitative findings demonstrated that participants perceived benefits for work–life integration and decreased work stress and work overload, which participants attributed to the program. Conclusions: Findings suggest that reduced work schedules may be associated with differences in employee wellbeing over the study period, particularly reduced work overload.

## 1. Introduction

Employees in public housing organizations face a demanding combination of workplace stressors (e.g., heavy workload, time pressures and interpersonal challenges [1]) while serving an economically vulnerable population under conditions of frequent resource constraint. The emotional labor requirements of this work, including the expectation to project positive attitudes and behaviors towards clients regardless of one’s internal state, add a further layer of demand that is characteristic of public sector roles more broadly [2]. Despite this burden, public housing workers have received limited attention in occupational health research, and the organizational conditions that support their wellbeing remain poorly understood. Two theoretical frameworks help explain these challenges. The organizational stress framework conceptualizes workplace wellbeing in terms of work stressors (tasks and role), moderators of stress (individual differences and social support), and consequences of stress, including burnout, depression, and heart disease [3]. The Job Demands–Resources (JD-R) model offers a complementary perspective, proposing that employee wellbeing depends on the balance between the demands placed on workers, including workload, time pressure, and emotional labor requirements, and the resources available to them, such as autonomy, collegial support, and opportunities for recovery [4]. When demands chronically outweigh available resources, employees are at increased risk for stress, burnout, and other negative impacts on their health. Within the JD-R framework, job engagement and supportive work culture can be understood as indicators of the organizational resources available to employees: job engagement reflects the motivational pathway through which resources such as autonomy, recovery time, and social support foster positive investment in work, while supportive work culture reflects the broader relational climate that supplies employees with resources central to sustaining wellbeing. Examining these positive dimensions alongside strain-related outcomes allows this study to assess not only whether the reduced work schedule reduced demand-related strain, but also whether it functioned as a resource that fostered engagement and a supportive workplace culture. These frameworks suggest that interventions that reduce demands or increase resources available to workers may improve wellbeing in high-demand public sector contexts.

One organizational response to workplace stress that has gained considerable traction in recent years is the reduced work schedule, typically referring to a 32 h, four-day workweek rather than the standard 40 h, five-day workweek, with no reduction in pay. Within the JD-R framework, reduced work schedules can be understood as an organizational resource. As the programs create protected time away from work, they provide employees with greater opportunity for recovery, restoration, and management of non-work responsibilities. The concept has a long history in the United States, dating to the 1950s and gaining increased popularity in the 1970s [5]. While interest faded in subsequent decades, reduced work schedules have seen renewed adoption as workplaces have sought to improve worker wellbeing and organizational efficiency, a trend accelerated by the COVID-19 pandemic and the wider normalization of flexible work arrangements [6]. The program evaluated in this study followed a related but distinct model: a voluntary 36 h reduced schedule with flexible timing of the reduced hours, rather than a fixed 32 h, four-day workweek. Because most existing research has focused on the latter, findings from four-day workweek studies discussed below may not generalize directly to more flexible reduced-schedule models such as the one examined here.

Early research on reduced work schedules suggests promising effects. Studies have linked shorter workweeks to lower employee burnout [7], improved work–life balance [5,7,8], greater job satisfaction [5,7,8], and increased productivity [8,9]. A large-scale international trial involving nearly 3000 employees across 141 organizations found improvements in burnout, job satisfaction, mental health, and physical health following implementation of a four-day workweek, with gains partially attributable to reduced fatigue and improved sleep [9]. A longitudinal panel study examining a single-organization working time reduction similarly found that reduced hours had the largest positive impact on work–family conflict among all wellbeing outcomes examined, with increased leisure time playing a key mediating role [10].

However, significant gaps and mixed evidence remain, and potential challenges to realizing these positive outcomes include scheduling conflicts, intensified monitoring and pressure from management, and the risk of exacerbating existing inequalities based on gender and work sector [5,11]. Questions have been raised about the sustainability of benefits over time, with some evidence of diminishing positive returns as program novelty fades [5]. There is also a gap in understanding how reduced work schedule programs operate at the organizational level and how their effects vary across role types and industries [8]. Most existing studies have relied on short follow-up periods and single methods of data collection, limiting the conclusions that can be drawn about sustained impacts and the mechanisms underlying them.

This study sought to address these gaps by evaluating associations between voluntary participation in a reduced work schedule program and employee wellbeing over 18 months at a public housing authority. Using a convergent mixed-methods design integrating longitudinal survey data with focus group findings, we examined whether program participants and non-participants differed in work stress, work-to-non-work conflict, work overload, job engagement, and supportive work culture across three timepoints. By combining quantitative and qualitative evidence in a population that has been largely absent from this literature, this study contributes both to the evidence base on the long-term effectiveness of reduced work schedule programs and to understanding of the contextual and role-based factors that shape their impact.

## 2. Materials and Methods

### 2.1. Study Design

We used a convergent mixed-methods design [12] to assess employee wellbeing in the context of a reduced work schedule. Quantitative data were collected through online surveys administered in February 2024 (T1), September 2024 (T2), and February 2025 (T3). A notable design limitation was the absence of a pre-implementation baseline because the reduced work schedule had already been implemented by the organization before data collection began. Thus, the analyses assess differences by participation status across post-implementation waves rather than changes attributable to program implementation. Qualitative data were collected through in-person focus groups conducted in March 2024. The study team integrated the survey and focus group findings during analysis, with integrated findings presented in the Discussion.

### 2.2. Study Setting, Participants, and Data Collection

We partnered with a Colorado-based public housing authority organization for this study. In fall 2023, the organization implemented a pilot program utilizing a reduced work schedule, lowering the number of weekly hours for a full-time employee from 40 or more hours per week to 36 h a week without a reduction in pay. The organization allowed all employees to opt in to the program once they had worked at the organization for at least six months. Rather than a fixed schedule reduction, the program allowed employees and work groups to select when their reduced hours occurred, providing flexibility in how the shortened schedule was structured across teams. The organization actively promoted employee wellbeing through its benefits program and other programmatic offerings such as mindfulness practices and wellbeing fairs. The leaders at the organization added the reduced work schedule program with the goals of increasing job engagement and a supportive work culture and reducing work stress, work-to-non-work conflict, and work overload among employees. In winter 2024, the organization reached out to the study team to evaluate the program to better understand the impacts of the program on aspects of employees’ wellbeing. All employees (*n* = 133) in the public housing authority were eligible to participate in the study, which was voluntary and did not collect identifiers.

For the surveys, the total number of respondents at each timepoint was similar (T1: 66; T2: 65; T3: 65). Employees transitioned between participation statuses during the study period (participating in the reduced work schedule and not participating; hereafter participants and non-participants, respectively). As a result, the composition of each group was not identical across waves.

For the focus groups, 30 employees participated in four group discussions conducted in March 2024, approximately six months after program implementation and concurrent with the first survey timepoint. Group size ranged from four to 10 participants each. Two focus groups were conducted with employees enrolled in the reduced work schedule who were able to fully use the shortened schedule; one focus group was conducted with employees enrolled in the program who were not able to fully use the shortened schedule regularly; and a final focus group was conducted with employees who did not participate in the program (i.e., non-participants). Focus group guides included open-ended questions about employees’ general experiences at the organization, their specific experiences with the reduced work schedule program, and their perceptions of the impacts of the reduced work schedule program. Focus groups were facilitated by one member of the study team, while another took detailed notes. Each focus group lasted one hour. Focus group participants verbally consented to participate in the group discussions.

The Colorado State University Institutional Review Board reviewed the protocol for the evaluation project (#5303) and determined it was not human subjects research due to its primary function as program evaluation.

#### Survey Measures

Wellbeing outcomes were assessed using measures from the NIOSH WellBQ (National Institute for Occupational Safety and Health [NIOSH]) [13], a validated instrument designed to capture worker wellbeing as a multidimensional construct, incorporating aspects of work experience, organizational context, and functioning both within and outside of work. Wellbeing outcomes in the present study included work stress (level of stress at work), job engagement (motivational-affective state: e.g., reporting work as inspiring), supportive work culture (perceived respect, fairness, and related climate items), work overload (not having enough time to get everything done), and work-to-non-work conflict (frequency with which job demands interfere with personal life). These outcomes were selected based on their overlap with common strains in reduced work schedule literature (e.g., work–life balance, stress/burnout, workload) [14]. In addition to these indicators of strain, the study assessed positive and organizational dimensions of wellbeing (i.e., job engagement and supportive work culture) to assess impacts on employee functioning and workplace context.

Work stress, work-to-non-work conflict, and job engagement were measured on a 7-point scale with higher scores reflecting more stress, work-to-non-work conflict, and job engagement. Supportive work culture and work overload were measured on a 4-point scale with higher scores reflecting a more supportive work culture and feelings of more work overload. Work stress, work-to-non-work conflict, and work overload were assessed using single-item measures, whereas job engagement and supportive work culture were assessed using multi-item scales. Internal consistency was acceptable for multi-item scales across timepoints (job engagement α = 0.75–0.82; supportive work culture α = 0.86–0.92). Other constructs were assessed using single-item measures, for which internal consistency estimates are not applicable [15].

### 2.3. Data Analysis

Linear mixed-effects models (LMMs) were used to examine differences across three timepoints, corresponding to 6, 12, and 18 months post-implementation, and differences associated with program participation. The sample had a partially overlapping longitudinal structure, as some employees contributed observations at multiple waves while others participated at only one timepoint. LMMs were therefore selected to account for the correlation among repeated observations from the same employee and to accommodate the unbalanced number of observations across individuals and timepoints under a missing-at-random assumption [16]. This approach is preferred over traditional linear models when some observations are clustered within individuals and respondents have incomplete survey data [17]. Models were estimated using all available observations, allowing employees to contribute data even if they did not complete every survey wave. Consequently, timepoint effects represent average differences across survey waves based on both repeated and non-repeated observations and should not be interpreted exclusively as within-person change.

Separate models were fitted for each outcome: work stress, job engagement, supportive work culture, work-to-non-work conflict, and work overload. Fixed effects included program participation, timepoint, and the interaction between participation and timepoint. Program participation was modeled as a time-varying predictor based on each employee’s enrollment status at the corresponding survey wave rather than their initial enrollment status. Employees who entered or left the program could therefore contribute observations to both participation groups across the study period. Random intercepts for employee ID were included to account for the correlation among repeated observations contributed by the same employee.

Analyses were conducted in R using RStudio version 2026.01.1 (Boston, MA, USA) [18], and LMMs were estimated using restricted maximum likelihood. Fixed effects were evaluated using Type III *F*-tests with Satterthwaite degrees of freedom, followed by pairwise comparisons where appropriate. Approximate partial eta squared values were calculated from the *F* statistics and degrees of freedom to quantify the magnitude of participation effects. Statistical significance was evaluated at α = 0.05, and Holm-adjusted *p* values were calculated across the five participation effects to control the familywise Type I error rate while retaining greater power than the standard Bonferroni correction [19]. Additionally, model assumptions were assessed using residual diagnostic plots. Log transformations were applied to job engagement, supportive work culture, and work-to-non-work conflict to address violations of normality and heteroskedasticity, whereas work stress and work overload were analyzed on their original scales. Estimated marginal means for transformed outcomes were back-transformed to the original response scales for presentation. Simulation-based observed-power analyses were conducted for the participation effect in each mixed-effects model using 1000 simulations.

The qualitative analysis consisted of thematic analysis using a deductive approach [20] of detailed focus group notes. Primary codes were derived from the five wellbeing outcome variables assessed in the quantitative component of the study: work-to-non-work conflict, work stress, work overload, supportive work culture, and job engagement. Data were additionally coded for employee role, with two categories distinguished: client-facing and non-client-facing. Two research team members independently coded two of the focus group notes using Dedoose version 10.0.59, Manhattan Beach, CA, USA [21]; then, codes were reviewed and reconciled through discussion to reach consensus before the final focus groups were coded and themes were created. An initial inter-rater reliability test was conducted using Dedoose’s Training Center feature, which calculates code-specific and pooled Cohen’s kappa statistics [22] using a pooling method appropriate for summarizing agreement across multiple codes [23]. This test yielded a pooled kappa of 0.74, indicating good agreement [24]; all codes exceeded a kappa of 0.80 except work stress (kappa = 0.54). Coders met to review and reconcile all work stress code applications before independently coding the remaining two focus groups.

## 3. Results

### 3.1. Descriptive Statistics

Respondents had diverse job roles at the organization, with the majority in client-facing roles (see Table 1), which included job roles in property management, rental assistance, maintenance, and resident services. The majority of respondents worked at the organization for 1–5 years (57%) or more (34%), with only 9% of all respondents new to the organization (less than 1 year). Respondent characteristics are summarized by reduced work schedule program enrollment at the initial timepoint in Table 1. Sample sizes by enrollment status and timepoint are presented in Table 2. Of the 133 unique employees, 85 (64%) completed one survey wave, 33 (25%) completed two waves, and 15 (11%) completed all three waves, resulting in a partially overlapping sample across timepoints. Most respondents were enrolled in the reduced work schedule program at each wave (T1: 85%; T2: 72%; T3: 80%). Among the 30 employees observed at both T1 and T2, seven (23%) changed enrollment status, including three who entered the program and four who moved from enrolled to not enrolled. Among the 24 employees observed at both T2 and T3, seven (29%) changed status, including four who entered the program and three who moved from enrolled to not enrolled.

### 3.2. Wellbeing Outcomes

Descriptive statistics for each wellbeing outcome across three timepoints by program participation status are presented in Table 2. Participants in the reduced work schedule program reported lower work stress, work overload, and work-to-non-work conflict relative to non-participants, while levels of job engagement and supportive work culture were comparable across groups. The gap in work overload was particularly pronounced, with participants reporting a mean of 2.12 (SD = 0.86) at T1 compared to 2.80 (SD = 1.14) among non-participants, widening to 1.91 (SD = 0.93) versus 3.00 (SD = 0.91) by T3.

Linear mixed-effects models were conducted to evaluate these patterns, with results presented in Table 3. Participation-by-timepoint interactions were tested in all models, but none reached statistical significance, indicating that the analyses did not detect differential change between groups across the observed waves. Given the small non-participant group and limited power to detect interaction effects, these findings should not be interpreted as evidence that group differences were stable or equivalent over time. After Holm correction across the five participation main-effect tests, participation remained associated with lower work overload, *F*(1, 193.64) = 17.88, unadjusted *p* < 0.001, Holm-adjusted *p* < 0.005, partial ηp^2^ = 0.085. Although participants also reported lower work-to-non-work conflict, *F*(1, 191.41) = 4.21, unadjusted *p* = 0.04, partial ηp^2^ = 0.022, and somewhat lower work stress, *F*(1, 189.58) = 3.87, unadjusted *p* = 0.05, partial ηp^2^ = 0.020, these differences did not remain statistically significant after correction. Participation effects for supportive work culture and job engagement were not statistically significant. There was little evidence of change in wellbeing indicators across the observed timepoints. Simulation-based observed-power estimates varied substantially across outcomes, ranging from 4.8% for job engagement and 6.3% for supportive work culture to 47.6% for work-to-non-work conflict, 50.0% for work stress, and 98.7% for work overload. These results indicated high sensitivity to detect the observed work-overload effect but limited sensitivity for effects of the magnitude observed for the other outcomes.

### 3.3. Thematic Findings

Deductive thematic analysis of the four focus groups resulted in five themes aligned with the wellbeing outcome variables: work-to-non-work conflict, work stress, work overload, supportive work culture, and job engagement. Table 4 provides descriptions and illustrative quotes for each theme. Because focus groups were conducted once, concurrent with the initial survey timepoint, these qualitative findings reflect employee perceptions shortly after program implementation and do not by themselves indicate whether perceptions changed over the 18-month study period.

Divergent views by role emerged across these themes, highlighting the complexity of the program’s impact. Client-facing employees and those in leadership positions described challenges in utilizing the program fully due to heavy workload demands, which often limited their ability to reduce their work hours. In contrast, employees in other roles described experiencing substantial benefits, including improved work–life balance and reduced work stress, emphasizing the role-specific factors influencing perceptions of the program.

#### 3.3.1. Work-to-Non-Work Conflict

Participants who were able to use the program consistently identified reduced conflict between work and non-work life as its most meaningful benefit. The additional time outside of work enabled participants to fulfill family responsibilities, prioritize personal health, and engage in other aspects of life outside of work. One non-client-facing participant described how the program positively impacted their family routine, saying, “I get to walk my daughter to school on Fridays with the reduced schedule, which increases my exercise and my family time.”

#### 3.3.2. Work Stress

Qualitative findings indicated that the relationship between the reduced work schedule and work stress varied by role and by the extent to which employees were able to use the program. Client-facing employees described how client needs often did not align with reduced availability, which in some cases led to compensatory overwork and heightened guilt about leaving tasks unfinished. For example, one client-facing participant said, “After I deal with hard clients, I still feel the stress after de-escalating, and it feels like there are not ways or time to debrief after the situation.”

In contrast, employees who fully used the program reported that time away from work allowed them to rest, recharge, and return feeling ready to engage, contributing to a more sustainable experience of work. As one non-client-facing participant said, “The reduced work schedule can allow for better mental health, more time off, which is something I look forward to and makes work more peaceful.”

#### 3.3.3. Work Overload

Qualitative findings on work overload revealed two distinct patterns based on role type. For many participants, the program functioned as a motivating structure that encouraged efficiency and helped them make the most of their working hours, which participants associated with reduced work overload. One non-client-facing participant said, “Reduced hours are my motivation to get things done knowing that you’re on more of a time crunch”.

In contrast, client-facing employees and those in leadership positions faced greater challenges in utilizing the program fully due to heavy and continuous workloads. These employees described feeling unable to step away, with one participant in a leadership position saying, “Those in positions of leadership haven’t been able to take the full amount of time off. Constant and overwhelming amount of work for those who are in leadership”. Another client-facing participant described how when they tried to use the reduced schedule, they ended up working longer hours later, saying, “I took off one day, and then worked 12 h on the weekend. I have too much to do to take time off”.

#### 3.3.4. Supportive Work Culture

The reduced work schedule program reinforced employees’ perceptions that the organization valued them as whole people. Participants who were able to fully use the program viewed it as a tangible expression of a supportive workplace culture. As one non-client-facing participant said, “We’re treated as human beings rather than just employees”.

Conversely, employees who felt unable to use the program fully, particularly those in understaffed units, expressed feelings of unfairness and perceived that their concerns about workload and staffing had not been adequately addressed. One client-facing participant said, “There are feelings of unfairness for some employees because they are picking up others’ slack”.

#### 3.3.5. Job Engagement

Employees across both participant and non-participant groups expressed a strong sense of connection to the organization’s mission, describing their work as meaningful and a source of pride. High job engagement appeared to be a characteristic of the organization rather than solely the result of program participation. Participants reported that the reduced work schedule enabled them to focus and prioritize tasks more effectively, which they described as increasing productivity and their ability to better serve the mission. As one non-client-facing participant said, “Interruptions are low during the quiet times, allows for productive time during the day, able to identify what needs to be focused on”. Another non-client-facing participant described how the program improved their engagement and efficiency, saying, “I am more productive at work with the reduced schedule”.

## 4. Discussion

This study evaluated the association between participation in a voluntary reduced work schedule program and employee wellbeing at a public housing authority using a convergent mixed-methods design [12]. After Holm adjustment for multiple testing, participation was associated with lower work overload, whereas the observed differences in work stress and work-to-non-work conflict did not remain statistically significant. The absence of significant participation-by-timepoint interactions should not be interpreted as evidence of sustained program effects because the study lacked a pre-implementation baseline and had limited power to detect differential change over time. Qualitative findings from focus groups conducted shortly after the initial timepoint mostly complemented these patterns while adding important nuance about the role of job type in shaping employees’ experiences of the program. Taken together, these findings contribute to a growing body of evidence supporting the partial wellbeing benefits of reduced work schedules [5,7,8,9] while highlighting conditions that shape whether those benefits are equitably realized.

### 4.1. Integration of Quantitative and Qualitative Findings

Findings for work overload converged across methods (see Table 5), though with important qualifications. Quantitatively, participants reported significantly lower work overload than non-participants across all timepoints, a finding that contrasts with patterns commonly reported in reduced work schedule research, where employees often absorb the same workload into fewer hours, resulting in intensified felt overload [14]. Qualitative data help explain this divergence. Participants described how having less time available encouraged more deliberate prioritization of tasks rather than simply working at greater intensity, suggesting that the structure of reduced hours may function as an organizational tool for time management. This interpretation aligns with research on the productivity benefits of reduced schedules [9], where constraints on available work time may prompt employees to work more purposefully rather than simply harder. However, in the qualitative findings, client-facing employees and those in leadership roles described a different experience, reporting difficulty stepping away from work and, in some cases, overwork. The difference in experiences across roles described in the focus groups, which has been identified as an underexplored dimension in the reduced work schedule literature [8], may have lessened what could otherwise have been an even stronger quantitative effect and points to a limitation of voluntary programs applied across heterogeneous job demands.

Work-to-non-work conflict showed partial convergence across quantitative and qualitative findings, though the quantitative difference did not remain statistically significant after correction for multiple comparisons. Program participants reported lower conflict between work and personal life in the quantitative analyses (unadjusted *p* = 0.04), although this difference did not remain statistically significant after Holm correction; it was nonetheless the benefit most frequently and emphatically raised by focus group participants. Employees described having more time for family, personal health, and rest, outcomes that align with the existing reduced work schedule literature consistently linking shorter schedules to improved work–life balance [5,7,8]. This aligns with a 2024 longitudinal study of a single-organization working time reduction that found work–family conflict to be the wellbeing domain most impacted by reduced work hours, with employees attributing improvements largely to having sufficient leisure time [10]. This result is particularly relevant for a public housing workforce, where employees already navigate the emotional labor requirements inherent in client-facing roles [1,2]. Within the JD-R framework [4], time protected from work demands serves as a resource, replenishing the capacity for sustained performance and supporting wellbeing among workers facing consistently high demands.

Work stress showed a related but weaker pattern. Although the unadjusted quantitative main effect approached significance (unadjusted *p* = 0.05), it did not remain statistically significant after Holm correction, and the reported difference in work stress between participants and non-participants should be interpreted with caution. Focus group findings nonetheless echoed a similar pattern, with participants describing reduced work stress, which they attributed primarily to the reduced schedule enabling them to take time to rest and recover between work periods. This is consistent with the JD-R model’s conceptualization of recovery time as a job resource that buffers against the depletion of demands [4] and with research identifying reduced fatigue and improved sleep as key mechanisms through which four-day workweeks improve wellbeing [9]. It also aligns with theoretical frameworks linking insufficient recovery to long-term consequences such as burnout and diminished health [3]. Client-facing employees were again an exception, describing heightened stress when reduced availability conflicted with client needs and generated guilt about unfinished work. This pattern raises concerns shared in the existing literature about the potential for reduced schedule programs to exacerbate existing inequalities by role and sector [5,11] and underscores that wellbeing benefits may be most accessible to employees whose work responsibilities can be more clearly defined and completed within scheduled hours.

Supportive work culture and job engagement did not differ significantly between groups in quantitative analyses. For job engagement, estimated marginal means were almost identical across groups, indicating near-perfect equivalence rather than a borderline non-significant difference. This suggests that high job engagement was a stable organizational characteristic rather than a program effect, a pattern consistent with the focus group data in which employees across both groups expressed strong mission alignment and connection to their work. Program participants did describe using their time more efficiently and feeling more productive, which the qualitative data suggest may represent a qualitative shift in how engagement was expressed rather than its overall level.

For supportive work culture, the quantitative pattern was more complex: descriptive means at T2 and T3 showed non-enrolled employees scoring marginally higher than enrolled employees, a trend that runs counter to the qualitative narrative in which program participants described the reduced schedule as evidence of a supportive organizational culture. This represents a genuine divergence across methods rather than a measurement artifact. One explanation is that employees unable to use the program, particularly those in understaffed or client-facing roles, expressed feelings of unfairness that may have suppressed enrolled employees’ perceptions of support relative to non-participants who remained on standard schedules. It is also possible that the organizational commitment to employee wellbeing, reflected in programming beyond the reduced schedule alone, contributed to a generally positive culture perception across both groups regardless of participation status, limiting any program-specific differences.

Notably, the association between participation and lower work overload was observed consistently across all three timepoints spanning 18 months, with no evidence of the diminishing returns that have been raised as a concern in the existing literature [5]. Descriptive means suggest the gap between groups on work overload slightly widened over time, with enrolled employees’ overload decreasing from T1 to T3 while non-enrolled employees’ overload increased over the same period, though the time-by-group interaction did not reach statistical significance and should not be interpreted as evidence of a stable or sustained effect. The difference in work-to-non-work conflict, which did not remain statistically significant after Holm correction, should be interpreted with similar caution. These patterns may have meaningful implications for organizations weighing the long-term value of reduced work schedule implementation, though the absence of a pre-implementation baseline and the observational design of this study limit causal interpretation.

### 4.2. Implications for Practice

These findings suggest that reduced work schedules may be associated with differences in employee wellbeing, particularly lower work overload, over at least 18 months. For public sector organizations and nonprofits with similarly complex, demanding work, these results offer cautious encouragement. However, organizations should anticipate differential uptake and benefit based on job role. Within a JD-R framework, client-facing and leadership roles carry higher baseline demands that may require supplementary job resources, including additional staffing, explicit workload redistribution plans, or role-specific adaptations to the schedule structure, before a reduced schedule program can be equitably and fully utilized across the workforce [4]. Without these supports, voluntary programs risk generating perceived unfairness between employees who can and cannot practically participate, a concern raised directly by focus group participants and one that could erode the supportive work culture the program is intended to reinforce. This finding aligns with critiques of reduced work schedule programs as potentially inequitable when implemented without structural attention to differences in job demands and workload distribution [11]. Organizations may also find value in clearly communicating expectations around workload coverage during reduced hours to prevent the informal redistribution of work onto colleagues, a dynamic that can impact the wellbeing of those carrying extra load. More specifically, organizations considering similar programs may benefit from identifying roles where workload volume or client-facing demands make hours difficult to compress, establishing explicit coverage plans for client-facing staff during reduced-schedule hours, and monitoring whether employees are compensating by working additional hours outside their formal reduced schedule.

### 4.3. Strengths, Limitations, and Future Directions

A key strength of this study is its longitudinal design, which allowed the study team to assess whether program effects were sustained rather than temporary, a question that has received limited attention in the existing literature [5]. The convergent mixed-methods design enabled quantitative and qualitative findings to be examined together, producing a more complete interpretation of results than either method alone would have allowed. The use of the validated NIOSH WellBQ [13] provided a multidimensional assessment of wellbeing grounded in established occupational health frameworks.

Several limitations of the study should be considered when interpreting the findings. Because researchers were engaged to conduct the program evaluation after program implementation, there was no pre-implementation baseline assessment, which prevents direct causal inference about the program’s effects. Group composition shifted across timepoints as employees moved between participation statuses, which may have introduced variability in estimates. The small and uneven non-participant group across waves reduced precision and made estimates more sensitive to individual observations, limiting the robustness of conclusions and precluding role-stratified quantitative analyses, although role differences were a consistent and informative theme in the qualitative data. Focus groups were conducted at a single timepoint shortly after T1 and may not fully reflect how employee perceptions evolved over the 18-month study period. The linear mixed models included only participation status, time, and their interaction as fixed effects, without adjustment for potential confounding variables such as organizational tenure, objective workload, or team size, which may have influenced the observed associations. Additionally, focus group data were captured through detailed facilitator notes rather than audio recordings and verbatim transcripts, which may have resulted in some loss of contextual or verbatim detail relative to recorded data.

Finally, this study was conducted within a single organization, which limits generalizability. Several factors related to this organization and its reduced work schedule program may have impacted the results and need to be considered when interpreting the findings. The organization implemented additional health- and safety-oriented programs for employees across the evaluation period, making it difficult to isolate the effects of the reduced work schedule program from the organizational investment in employee wellbeing. The organization’s pre-existing commitment to employee wellbeing and positive work culture may have created conditions particularly conducive to the reduced work schedule program’s success. The reduced schedule program differed from the reduced work schedule models most examined in the literature. Rather than a fixed reduction to 32 h, employees and work groups participated in a 36 h variable schedule in which they could select when their reduced hours occurred. While this design offers greater flexibility and may better reflect how such programs are implemented in practice, it limits direct comparability with existing research and introduces variability in how the program was experienced by employees across roles and teams.

Future research should incorporate pre-implementation baseline measures and randomized or matched comparison designs where feasible to enable stronger causal inference. Examining how role type, workload volume, and staffing levels moderate program impact on wellbeing would be particularly valuable for understanding how to adapt reduced work schedules equitably across roles. Research at the organizational level, examining how program design, leadership support, and workload management policies shape employee outcomes, would also help address the gap in understanding how macro-level program features translate to individual wellbeing [8]. Longitudinal qualitative data collection across multiple timepoints would further enrich understanding of how employee experiences and perceptions of the program evolve over time.

## 5. Conclusions

This study found that voluntary reduced work schedule program participation was associated with differences in public housing employee wellbeing over 18 months. Program participants reported significantly lower work overload than non-participants; differences in work stress and work-to-non-work conflict were smaller and did not remain statistically significant after correction for multiple comparisons. There was no evidence that the magnitude of these differences changed across timepoints, although limited statistical power constrains interpretation of this finding as evidence of stability. Qualitative findings largely reinforced these results, illustrating how reclaimed time outside of work enabled employees to rest, attend to personal responsibilities, and return to work feeling ready to complete their demanding work. However, benefits were not uniformly experienced, as described in the focus groups. Client-facing employees and those in leadership roles faced barriers to fully utilizing the program. These findings suggest that distributing the wellbeing benefits of reduced work schedules equitably across a workforce requires attention to workload management and staffing conditions in addition to the scheduling changes.

## Figures and Tables

**Table 1:** Tenure and role by reduced work schedule program participation status at the initial timepoint.

Characteristic	Enrolled in Reduced Work Schedule (*n* = 50)	Not Enrolled in Reduced Work Schedule (*n* = 8)	All Respondents (*n* = 58)
Tenure			
Less than a year	8%	13%	9%
1–5 years	60%	38%	57%
6–10 years	12%	13%	12%
More than 10 years	20%	38%	22%
Role			
Client-facing	62%	88%	66%
Non-client-facing	38%	12%	34%

Note. Demographic characteristics are presented for the 58 Timepoint 1 respondents with complete tenure and role data.

**Table 2:** Means and standard deviations for wellbeing outcomes by reduced work schedule program enrollment status and timepoint.

	Timepoint 1 Mean (SD)		Timepoint 2 Mean (SD)		Timepoint 3 Mean (SD)	
	Enrolled (*n* = 56)	Not Enrolled (*n* = 10)	Enrolled (*n* = 47)	Not Enrolled (*n* = 18)	Enrolled (*n* = 52)	Not Enrolled (*n* = 13)
Work-to-Non-work Conflict	3.67 (1.41)	4.30 (1.89)	3.63 (1.32)	4.37 (1.54)	3.49 (1.44)	4.08 (1.80)
Work Stress	4.84 (1.24)	5.40 (1.17)	4.92 (1.38)	5.21 (1.13)	4.87 (1.33)	5.38 (1.39)
Work Overload	2.12 (0.86)	2.80 (1.14)	2.12 (0.97)	2.68 (0.82)	1.91 (0.93)	3.00 (0.91)
Supportive Work Culture	3.30 (0.64)	3.20 (0.90)	2.99 (0.78)	3.15 (0.78)	3.18 (0.77)	3.28 (0.70)
Job Engagement	5.79 (0.73)	5.77 (1.20)	5.63 (0.96)	5.64 (0.95)	5.54 (1.16)	5.56 (0.82)

Note. Work stress, work-to-non-work conflict, and job engagement were measured on a 7-point scale with higher scores reflecting more stress, work-to-non-work conflict, and job engagement; supportive work culture and work overload were measured on a 4-point scale with higher scores reflecting a more supportive work culture and feelings of more work overload, respectively.

**Table 3:** Main effect of reduced work schedule participation on wellbeing outcomes.

Wellbeing Outcome	Enrolled in Reduced Work Schedule Program	Estimated Mean (SE)	Effect	*p*-Value	Holm’s Adjusted *p*	Partial ηp^2^
Work-to-Non-work Conflict	Enrolled	3.31 (0.12)	F(1, 191.41) = 4.21	0.04	0.16	0.022
	Not Enrolled	3.91 (0.27)				
Work Stress	Enrolled	4.89 (0.11)	F(1, 189.58) = 3.87	0.05	0.16	0.020
	Not Enrolled	5.31 (0.20)				
Work Overload	Enrolled	2.07 (0.07)	F(1, 193.64) = 17.88	<0.001	<0.005	0.085
	Not Enrolled	2.73 (0.14)				
Supportive Work Culture	Enrolled	3.03 (0.08)	F(1, 124.64) = 0.37	0.55	1.00	0.002
	Not Enrolled	3.06 (0.12)				
Job Engagement	Enrolled	5.58 (0.05)	F(1, 195.99) ≈ 0.00	0.99	1.00	<0.001
	Not Enrolled	5.58 (0.03)				

Note. Estimated means represent model-adjusted marginal means from linear mixed-effects models, accounting for timepoint.

**Table 4:** Thematic findings for the five themes with illustrative quotes.

Themes	Description	Illustrative Quotes (Employee Role)
Work-to-Non-work Conflict	Participants identified reduced work-to-non-work conflict as the program’s greatest benefit, using reclaimed time to support family, personal health, and life outside of work.	“I found that the extra hours were beneficial to life outside of work, and it allows for time with family and grounding myself.” (Non-client-facing)
		“I feel like I can do things in my life without having to ask for time off. Now I get to save PTO [paid time off] for things like trips.” (Client-facing)
Work Stress	Employees who used the program fully reported being able to rest, have less stress, and return to work feeling recharged.	“The program helps me ease into the weekend with less work stress.” (Non-client-facing)
	Client-facing employees sometimes described experiencing heightened stress due to misaligned client demands and the reduced schedule.	“It’s hard to stop at noon when there’s so much more to do [for clients] and not feel guilty about it.” (Client-facing)
Work Overload	The program motivated efficiency and reduced work overload for many participants.	“My work is less consuming.” (Non-client-facing)
	Client-facing employees and those in leadership found it difficult to fully utilize the reduced hours due to heavy workloads.	“Managers have a heavier workload. Since there is not a clear cut off for when managers are done working, it feels less feasible [to fully use the program].” (Leadership)
Supportive Work Culture	Employees who participated in the program viewed it as evidence of a supportive culture.	“[The program is part of our] intentional work culture aimed at making staff safe and successful.” (Non-client-facing)
	Those unable to use it described feelings of unfairness, which impacted their perceptions of supportive work culture.	“The program feels like cutting jobs to try to save money and putting the work of many on one person.” (Client-facing)
Job Engagement	Regardless of their participation in the program, employees across both groups felt inspired by their work and connected to the organization’s mission.	“No matter the department we agree with the mission.” (Client-facing)
	Program participants described how the program helped them focus and work more productively.	“I make the most out of the time that I have when I’m working.” (Non-client-facing)

**Table 5:** Integration of quantitative and qualitative findings across wellbeing outcomes.

Outcome	Quantitative Finding (Holm-Adjusted)	Qualitative Finding	Convergence
Work Overload	Significantly lower among participants (Holm-adjusted *p* < 0.005)	Participants described more deliberate prioritization and reduced overload; client-facing and leadership roles described continued difficulty stepping away from work	Convergent, with an important qualification by role
Work-to-Non-work Conflict	Lower among participants; did not remain significant after Holm correction	Most frequently and emphatically cited benefit across focus groups	Partial convergence: qualitative pattern stronger than quantitative evidence
Work Stress	Marginally lower among participants; did not remain significant after Holm correction	Participants described reduced stress from recovery time; client-facing employees described heightened stress from misaligned client demands	Partial convergence: qualitative pattern stronger than quantitative evidence and diverges by role
Supportive Work Culture	No significant difference between groups	Enrolled employees described the program as evidence of a supportive culture; some non-enrolled employees described feelings of unfairness	Divergent
Job Engagement	No significant difference between groups (near-identical estimated means)	High engagement described across both groups; participants described more focused productivity	Convergent (both indicate no program-specific difference)

## Data Availability

The raw data on the employees of the organization that piloted the shorter workweek, as well as the aggregated data, are protected and shared only with the study team. The organization only consented to the data being used by the study team. Upon request, our syntaxes of quantitative analyses can be made available. Please e-mail morgan.valley@colostate.edu.

## Abbreviations

The following abbreviations are used in this manuscript:

JD-R	Job Demands–Resources (model)
LMM	Linear mixed-effects model
NIOSH	National Institute for Occupational Safety and Health
PTO	Paid time off
REML	Restricted maximum likelihood
SD	Standard deviation
SE	Standard error
T1, T2, T3	Timepoint 1, Timepoint 2, Timepoint 3 (survey waves)
WellBQ	Worker Well-Being Questionnaire
